# Induced autopolyploidy—a promising approach for enhanced biosynthesis of plant secondary metabolites: an insight

**DOI:** 10.1186/s43141-020-00109-8

**Published:** 2021-01-08

**Authors:** Saikat Gantait, Eashan Mukherjee

**Affiliations:** Crop Research Unit (Genetics and Plant Breeding), Bidhan Chandra Krishi Viswavidyalaya, Mohanpur, Nadia, West Bengal 741252 India

**Keywords:** Antimitotic agents, Autotetraploid, Biosynthetic pathway, Colchicine, Oryzalin

## Abstract

**Background:**

Induced polyploidy serves as an efficient approach in extricating genetic potential of cells. During polyploidization, multiple sets of chromosomes are derived from the same organism resulting in the development of an autopolyploid. Alterations owing to artificially induced polyploidy level significantly influence internal homeostatic condition of resultant cells.

**Main text:**

Induced autopolyploidy transpires as a result of an increase in the size of genome without any change in elementary genetic material. Such autopolyploidy, artificially induced via application of antimitotic agents, brings about a lot of beneficial changes in plants, coupled with very few detrimental effects. Induced autopolyploids exhibit superior adaptability, endurance to biotic and abiotic stresses, longer reproductive period and enzyme diversity coupled with enhanced rate of photosynthesis and gene action in comparison to their diploid counterparts. However, reduced rate of transpiration and growth, delay in flowering are some of the demerits of autopolyploids. Inspite of these slight unfavourable outcomes, induced autopolyploidization has been utilized in an array of instances wherein genetic improvement of plant species is concerned, since this technique usually boosts the biomass of concerned economic parts of a plant. In other way, it is also evident that multiplication of genome bestows enhanced production of secondary metabolites, which has contributed to a significant commercial value addition especially for plants with medicinal importance, in particular.

**Conclusion:**

This review makes an attempt to explore the system and success of antimitotic agents vis-à-vis artificial autopolyploidization, interfered with the biosynthesis-cum-production of secondary metabolites having cutting-edge pharmaceutical importance.

## Background

Induced polyploidy serves as an effective tool in extricating a cell’s genetic potential. During polyploidization, when multiple sets of chromosomes are derived from the same organism, it results into the development of autopolyploidy. Induced multiple ploidy level affects a cell’s internal homeostatic condition. This may lead to meiotic instabilities, undesirably magnified linkage effects, chimeral tissues [1], poor sexual reproduction and other epigenetic changes in plants. Such aberrational problems are often associated with triploids and mixoploids whereby there persists a problem to pair chromosomes during meiosis. Despite these encumbrances, induced polyploidy provides several mileages in crop research and improvement. Firstly, duplication of the same genomic constituents produces genetically true-to-type individuals [2]. Genome re-duplication characterizes increased plant vigour and productivity through improved cellular activity status increasing cell size, stomatal size and number, nuclear size and activity [2, 3], gene interaction and expression, transcriptome output, coordinated post-translational gene regulation resulting in increased metabolism, and henceforth higher production of alkaloids, flavones, phenolics, collectively the secondary metabolites [4, 5]. As a consequence, both the quality and quantity of the secondary metabolite production receives a massive boost [6]. Such improvement could be obtained in proportionate terms across triploids, tetraploids and at other higher ploidy levels due to underlying multiple genetic dose effects [7]. However, such manipulative changes are also subject to genotype specificities.

Even though two review articles were published most recently on artificial polyploidization of medicinal and aromatic plants [5, 6], yet neither of them emphasized on the enhancement of secondary metabolite production precisely; in spite of the fact that genetic improvement of medicinal and aromatic plants focuses chiefly on quantitative development of essential oils and secondary metabolites.

Nearly about three decades earlier, the utility of induced polyploids for enhancing the productivity of secondary metabolites was highlighted [8]. Yet, it took more than 15 years to commence the all-encompassing usage of this technology, wherein starting from 2011 to 2020, more than 60 research articles have been published focusing primarily on the use of antimitotic agents (e.g. colchicine, oryzalin and/or trifluralin, etc.) in order to multiply the ploidy level for attainment of elevated production of secondary metabolites (Table 1). Meanwhile, to the best of our knowledge, merely six results were reported on this concerned research topic in those 15 years (commencing from 1996 to 2010) [73–78]. It is noteworthy to mention that an array (~ 100) of successful attempts were reported [5, 6] on induction of polyploidy in medicinal and aromatic plants; however, ~ 62 of such reports were related to elevation of secondary metabolites production only.

**Table 1:** Antimitotic agent(s) and their influence on autopolyploidization and subsequent secondary metabolites production (arranged in chronological order)

Plant spp.	Treatment condition	Treated plant part	Type, dosage and exposure duration of antimitotic agent(s)	Resultant ploidy level	Effect on secondary metabolite production	Reference
*Catharanthus roseus* Don.	Immersed in colchicine (dissolved in ethanol) soln.	Seed	Colchicine; 0.2%; 24 h	Diploid➔Tetraploid (2n=4x=32)	Increase in terpenoid indole alkaloids (2–3-fold)	[9]
*Centella asiatica* (L.)	Soaked in colchicine solution	Shoot tip	Colchicine; 0.050–0.200%; 12–24 h	Diploid➔Tetraploid (2n=4x=36)	Non-significant increase of 11% in total triterpenes production	[10]
*Catharanthus roseus*	NM	Shooty teratoma	Colchicine: 0.01%; 24–48 h	Diploid➔Tetraploid (2n=4x=32)	2-fold increment in vincristine production	[11]
*Solanum commersonii*	Dipped in sterile and agitated (100 rpm at r.t.) H_2_O containing oryzalin (40 mg/l dissolved in 1 ml of DMSO)	Shoot tip	Oryzalin; 10 mg /l; 24 h	Diploid➔Tetraploid (2n=4x=48)	Higher phenylpropanoid content (approx. 2-fold)	[12]
*Solanum bulbocastanum*	Dipped in sterile and agitated (100 rpm at r.t.) H_2_O containing oryzalin (40 mg/l dissolved in 1 ml of DMSO)	Shoot tip	Oryzalin; 10 mg /l; 24 h	Diploid➔Tetraploid (2n=4x=48)	Similar or lower phenylpropanoids and metabolites compared to diploids	[13]
*Rosa hybrida* cv Iceberg	NM	NM	Trifluralin; NM; NM	Triploid➔Hexaploid (2n=6x=42)	Increase in phenolic compounds	[14]
*Echinacea purpurea* (L.)	Colchicine soln. imbibition	Root tip	Colchicine; 0.25%; 24–72 h	Diploid➔Tetraploid (2n=4x=44)	Increase in cichoric acid (45%) and chlorogenic acid (71%)	[15]
*Stevia rebaudiana*	H_2_O imbibed seed➔ colchicine (dissolved in 1% DMSO) soln.	Seed	Colchicine; 0.6%; 24 h	Diploid➔Tetraploid (2n=4x=44)	Increase in stevioside (approx. 2.4%)	[16]
*Cichorium intybus* L	cotton covered meristem tip➔ 3 droplets/treatment➔ 4 times at treatment intervals	Shoot apical meristem	Colchicine; 0.05%; 8 h	Diploid➔Tetraploid (2n=4x=36)	1.9-fold increase in total phenolic; 10-fold increase in chlorogenic acid	[17]
*Allium sativum* L.	Soaked in basal media (liquid B5 + 2% DMSO + colchicine)	Stem disc	Colchicine; 0.5%; 36 h	Diploid➔tetraploid (2n=4x=32)	Increase in allicin conc. (30.7%)	[18]
*Pfaffia glomerata* (Spreng.) Pedersen	Basal media + colchicine	Nodal bud	Colchicine; 30 μM; 1 w	Diploid➔tetraploid (2n=4x=68)	Increase in β-ecdysone production (50%)	[19]
*Dracocephalum kotschyi* Boiss	Colchicine soln.➔ cotton ball method➔ SAM (2 leaf and 4 leaf stage)	Shoot apical meristem	Colchicine; 0.5%; 48 h	Diploid➔tetraploid (2n=4x=40)	Increase in methoxylated hydroxyflavones (approx. 19.41%)	[20]
*Centella asiatica* (L.)	Soaked in colchicine solution	Shoot tip	Colchicine; 0.050–0.200%; 12–24 h	Diploid➔Tetraploid (2n=4x=36)	Increase in total triterpenoid (approx. 30.5%)	[21]
*Echinacea purpurea* (L.) Moench	Shoot regeneration media + colchicine	Petiole	Colchicine; 30–240 mg/l for 30 d or 120 mg/l for 7, 14, 21 or 28 d	Diploid➔tetraploid (2n=4x=44)	Increase in caffeic acid and alkamides	[22]
*Nigella sativa* L.	Colchicine (dissolved in 2% DMSO) soln. treatment	Seed	Colchicine; 0.05%; 4 h	Diploid➔tetraploid (2n=4x=24)	Increase in thymoquinone conc. (46.3%)	[23]
*Stevia rebaudiana* Bertoni	NM	NM	Colchicine; NM; NM	Diploid➔tetraploid (2n=4x=44)	Increase in rebaudioside-A (6.21%) in tetraploid;	[24]
				Triploids and mixoploids	Increase in (13.50%) stevioside	
*Thymus persicus*	Immersed in colchicine soln. (dissolved in 2% DMSO)➔ sterile H_2_O washing	Shoot tip	Colchicine; 0.3%; 12 h	Diploid➔tetraploid (2n=4x=56)	Increase in betulinic acid (69.73%), oleanolic acid (42.76%), ursolic acid (140.67%)	[25]
*Stevia rebaudiana* Bertoni	Treatment in field condition	NM	Colchicine; 2.5%; NM	Diploid➔tetraploid (2n=4x=44)	Increase in rebaudioside-A (6.21%)	[26]
			Colchicine; 1.50%; NM	Mixoploid	Increase in steviosisde (13.50%)	
*Tetradenia riparia*	Submerged in colchicine soln.	Seed	Colchicine; 0.01 g/l; 24 h	Diploid➔tetraploid (2n=4x=)	Increase in essential oi production (3.5-fold)	[27]
*Echinacea purpurea* L.	MS + 0.4 mg/l BA + 0.01 mg/l NAA + Colchicine	Petiole	Colchicine; 100 mg/l; 37 d	Diploid➔tetraploid (2n=4x=44) Tetraploid➔octaploid (2n=8x=88)	Increase in cichoric acid content in octoploid (13.76 and 29.84 mg/g) and tetraploid (10.38 and 22.32 mg/g) than diploid plantlets (8.21 and 18.42 mg/g) aboveground and underground parts, respectively	[28]
*Miscanthus* × *giganteus*	NM	NM	Colchicine; NM; NM	Triploid➔hexaploid (2n=6x=114)	Lower phenolic compounds in hexaploids (1266 μg/g and 1349 μg/g) than triploid (1570 μg/g and 2268 μg/g)	[29]
*Lippia integrifolia*	(MS + 2.2 μM BA) + colchicine	Nodal segment	Colchicine; 0.01%; 15 d	Diploid➔tetraploid (2n=4x=72)	Increase in total monoterpene (9%)	[30]
*Pogostemon cablin*	MSL + colchicine➔ shaking at 100 rpm at 25°C	Cluster bud	Colchicine; 0.05%; 72 h	Tetraploid➔octaploid (2n=8x=128)	Increase in patchoulic alcohol (2.6-fold)	[31]
*Chamaemelum nobile* L.	MSL + 1 μM NAA + 0.5 μM BAP + Colchicine; 25 °C; Dark for 12 h	Callus	Colchicine; 0.1%; 12 h	Diploid➔tetraploid (2n=4x=36)	Increase in essential oil (2-fold)	[32]
*Bacopa monnieri*	Treated with colchicine (dissolved in 1% DMSO)	Leaf	Colchicine; 0.1%; 2 h	Diploid➔tetraploid (2n=4x=128)	Increase in bacoside (more than 4-fold)	[33]
*Capsicum frutescens* L.	Immersed in colchicine soln. under dark condition; shaken at 100 rpm	Seed	Colchicine; 300 mg/l; 6 h	Diploid➔tetraploid (2n=4x=48)	Increase in capsaicin (1.37-fold)	[34]
			Oryzalin; 30 mg/l; 6 h		Increase in capsaicin (1.36-fold)	
	Immersed in oryzalin soln. under dark condition shaken at 100 rpm					
*Aquilaria malaccensis* Lamarck	NM	Nodal segment	Trifluralin; 0.1 mM; 120 h	Diploid➔tetraploid (2n=4x=28)	Presence of sesquiterpene, α-guaiene (2.92%)	[35]
*Cannabis sativa* L.	Through micropipette ➔ 4 times at 6 h interval	Apical meristem	Colchicine; 0.2%; 24 h	Diploid➔tetraploid (2n=4x=40)	Increase in cannabinoid (1.2-fold)	[36]
			Colchicine; 0.1%; 24 h	Diploid➔mixoploid	Increase in cannabinoid (1.6-fold)	
*Anoectochilus formosanus* Hayata	MSL + colchicine	Nodal stem	Colchicine; 100 mg/l; 3 d	Diploid➔tetraploid (2n=4x=48)	Increase in total flavonoid (0.5-fold) and gastrodin (4-fold)	[37]
*Trachyspermum ammi* L.	MS + colchicine (Dissolved in distilled H_2_O + 2% DMSO)	Seed	Colchicine; 0.05%; 24 h	Diploid➔tetraploid (2n=4x=36)	Increase in thymol in tetraploid (69.2%) over diploids (49.67%)	[38]
*Linum album*	MS + 0.4 mg/l KIN + colchicine➔ shaking at 100 rpm	Nodal segment	Colchicine; 2.5 mM; 72 h	Diploid➔tetraploid (2n=4x=60)	Increase in total phenolics (1.8-and 2.1-fold) and flavonoids (1.55- and 1.53-fold) in regenerated stem and leaf, respectively	[39]
*Platycodon grandiflorum*	NM	Seedling	Colchicine; NM; NM	Diploid➔tetraploid (2n=4x=36)	Increase in metabolite content (NM)	[40]
*Dendrobium* hybrids	Suspension culture + colchicine/ Amiprophos-methyl	Protocorm like body	Colchicine; 0.050%; 3 d Amiprophos-methyl; 2.5 μM; 3 d	Diploid➔tetraploid (2n=4x=76)	Increase in shihundine and hircinol (NM)	[41]
*Sphagneticola calendulacea* (L.) Pruski	Incubation in MSL+ 2% DMSO + Colchicine➔ shaking (100 rpm) at 25 °C	Shoot tip	Colchicine; 0.05%; 24 h	Diploid➔tetraploid (2n=4x=100)	Increase in wedelolactone (1.66-fold)	[42]
*Stevia rebaudiana* Bertoni	Imbibed in colchicine soln. (dissolved in 2% DMSO) in dark	Seed	Colchicine; .01%; 24 h	Diploid➔tetraploid (2n=4x=44)	Increase in stevioside (2.5-fold) and rebaudioside-A (1.5-fold)	[43]
*Eclipta alba* (L.) Hassk	MSL+ colchicine + 2% DMSO➔ shaking at 100 rpm	Shoot tip	Colchicine; 0.1%; 24 h	Diploid➔tetraploid (2n=4x=44)	Increase in wedelolactone (approx. 3-fold)	[44]
*Stevia rebaudiana* Bertoni	Cotton plug method➔ colchicine-soaked cotton placed on bud	Axillary bud	Colchicine; 1.0%; 3 d	Diploid➔tetraploid (2n=4x=44)	Increase in stevioside (2.5-fold)	[45]
*Sophora tonkinensis* Gapnep	Immersed in colchicine soln.	Adventitious bud (from epicotyl)	Colchicine; 0.2%; 30 h	Diploid➔tetraploid (2n=4x=36)	Higher productivity of matrine and oxymatrine (1.31-fold)	[46]
*Andrographis paniculata*	Soaked in colchicine soln at 4 ± 1 °C➔ fresh colchicine soln. at 40 ± 1 °C	Seed	Colchicine;0.01%; 40 min & 20 min	Diploid➔tetraploid (2n=4x=100)	Increase in andrographolide (28%)	[47]
*Salvia miltiorrhiza*	MS + 0.5 mg/l TDZ + colchicine	Leaf segment	Colchicine; 0.5 mg/l; 3 w	Diploid➔tetraploid (2n=4x=32)	Increase in dihydrotanshinone and total tanshinones (approx. 1.5-fold)	[48]
*Bletilla striata* (Thunb.) Reichb.	MSL + colchicine; dark at 25 ± 2 °C	Seed	Colchicine; 0.1%; 7 d	Diploid➔tetraploid (2n=4x=64)	Increase in total phenolics (1.6 folds)	[49]
*Andrographis paniculata* (Burm. F.) wall. ex Nees	NM	NM	Colchicine; NM; NM	Diploid➔tetraploid (2n=4x=100)	Increase in leaf metabolite (NM)	[50]
*Stevia rebaudiana* (Bertoni)	Inoculation in colchicine soln.	Adventitious bud	Colchicine; 0.20%; 12 h	Diploid➔tetraploid (2n=4x=44)	Increase in steviol glycosides (approx. 1.27-fold)	[51]
*Datura stramonium* L.	Soaked in colchicine	Seed	Colchicine; 1%; 48 h	Diploid➔tetraploid (2n=4x=48)	Increase in alkaloids (7.25%)	[52]
*Eucommia* sp.	High temperature induction of 2n female parent➔ 2n female gamete × haploid pollen	Megaspore	High temperature; NM; NM	Diploid × haploid ➔ triploid (3n=51)	Increase in chlorogenic acid (10.20%), aucubin (1.34%), geniposide (118.22%), geniposidic acid (22.04%)	[53]
*Bacopa monnieri*	MSL + colchicine soln. ➔ shaking (100 rpm) at 25±2°C; 10 h PP; 20 μmol/m^2^ LI	Nodal segment	Colchicine; 0.5%; 48 h	Diploid➔tetraploid (2n=4x=128)	Increase in bacoside (2.3-fold)	[54]
*Cannabis sativa*	Dipped in MSL + oryzalin media (37.5 mM oryzalin dissolved in 80% ethanol)➔ tin foil cover of cultures➔ shaking (150 rpm)	Axillary bud	Oryzalin; 20–40 μM; 24 h	Diploid➔tetraploid (2n=4x=40)	Increase in cannabidiol (9%)	[55]
garlic	Imbibed in colchicine soln. (5 drops of 4% DMSO added)	Clove	Colchicine; 0.6%; 24 h	Diploid➔tetraploid (2n=4x=32)	Increase in diallyl disulfide (15.13%), allyl methyl trisulfide (24.61%) and diallyl trisulfide (15.57%)	[56]
*Physalis peruviana* L.	Soaking in colchicine soln.	Seed	Colchicine; 0.6%; 36 h	Diploid➔tetraploid (2n=4x=48)	Increase in total flavonoid (88.7%)	[57]
	Germinated seed with 2–3 mm long root in colchicine soln.		Colchicine; 0.6%; 24 h		Increase in total anthocyanin (9.1%)	
	Soaking in colchicine soln.		Colchicine; 0.6%; 24 h		Increase in total phenolic (NM)	
*Citrus limon* (L.) Osbeck	MS + colchicine; 25 ± 2 °C; 16 h PP; 50–60 μmol/m^2^/s LI	Seed with radicle	Colchicine; 0.025%; 24 h	Diploid➔tetraploid (2n=4x=36)	Increase in essential oil and limonene (7%)	[58]
*Eucalyptus polybractea* RT Baker	Solid medium M1 + colchicine; Dark; 25 °C; shaking at 40 rpm	Shoot tip	Colchicine; 0.5%; 4 d	Diploid➔tetraploid (2n=4x=44)	Increase in essential oil (3%)	[59]
*Plantago psyllium*	Colchicine treatment (dissolved in 2% DMSO)	Terminal bud	Colchicine; 0.5%; 24 h	Diploid➔tetraploid (2n=4x=24)	Increase in carotenoid (1.5-fold)	[60]
	NM		Trifluralin; 22.5%; 72 h		Increase in carotenoid (1.54-fold)	
*Plantago ovata*	Soaking in colchicine soln.	Terminal bud	Colchicine; 0.3%; 24 h Trifluralin; 22.5%; 72 h	Diploid➔tetraploid (2n=4x=16)	Increase in carotenoid content (approx. 1.83-fold)	[61]
*Thymus vulgaris* L.	Oryzalin (dissolved in 1% DMSO) treatment➔ sterile H_2_O washing➔ culture in MS	Nodal segment	Oryzalin; 80 μM; 24 h	Diploid➔tetraploid (2n=4x=60)	Essential oil content increase (from 0.81 to 1.19%); increase in thymol (18.01%) and carvacol (0.49%)	[62]
*Melissa officinalis* L.	Soaked in colchicine soln.	Seedling	Colchicine; 0.1%; 24 h	Diploid➔tetraploid (2n=4x=64)	Increase in carotenoid content (2.9-fold)	[63]
			Colchicine; 0.05%; 24 h		Increase in phenolic content (4 times)	
			Colchicine; 0.1%; 48 h		Increase in flavonoid content (4.93-fold)	
*Lippia alba*	Inoculation in colchicine +MS medium	Nodal segment	Colchicine; 0.2%; 72 h Colchicine; 0.2%; 4 h	Diploid➔tetraploid (2n=4x=60) Diploid➔mixoploid	Increase in linalool content; insignificant change in ctiral content	[64]
*Zingiber officinale* Roscoe cv. ‘Fengtou’ ginger	MSL + colchicine soln. ➔ shaking at 150 rpm at 23°C dark	Stem segment	Colchicine; 150mg/l; 7 d	Diploid➔ tetraploid (2n=4x=44)	Increase in carotenoids conc. (1.375-fold)	[65]
*Panax ginseng* Mayer	MSL + colchicine soln. ➔ shaking at 100rpm ➔ dark	Adventitious root	Colchicine; 100mg/l; 1 d	Tetraploid ➔ mutant (tetraploid)	Increase in ginsenoside production (4.85-fold)	[66]
*Stevia rebaudiana*	Soaked in colchicine soln.	Seed	Colchicine; 0.2%; 24 h	Diploid➔tetraploid (2n=4x=44)	Increase in rebaudioside-A content (2.84%)	[67]
*Artemisia cina*	Soaked in colchicine soln.	Shoot	Colchicine; 100 mg/l	Diploid➔ tetraploid (2n=4x=36)	Increase in quercetin content (1.34-fold); kaempferol content (1.47-fold)	[68]
*Artemisia cina*	NM	NM	Colchicine; NM; NM	Diploid➔ tetraploid (2n=4x=36)	Increase in artemisinin content (NM)	[69]
*Solanum tuberosum*	In vitro colchicine treatment	(*Solanum tuberosum* × *Solanum kurtzianum*)	Colchicine; NM; NM	Diploid➔tetraploid (2n=4x=48) (Allotetraploid)	Increase in sesquiterpenes (22-fold)	[70]
	Oryzalin treatment	Diploid line (*Solanum kurtzianum*)	Oryzalin; NM; NM	Diploid➔tetraploid (2n=4x=48) (Autotetraploid)	Increase in sesquiterpenes (6-fold)	

*2*-*IP* 2-isopentenyl adenine; *B5* Gamborg’s B5 media [71]; *BA* N^6^-benzyladenine; *d* days, *DMSO* dimethyl sulfoxide; *LI* light intensity; *M1* media [woody basal salt mixture (2.3 g/l) + Murashige and Skoog modified vitamins (1000x) (1 ml/l) + Sucrose (25 g/l) + 2IP (3 μM) + Agar (7 g/l)]; *m* months; *min* minutes; *MS* Murashige and Skoog [72]; *MSL* MS Liquid Media; *NAA* α-naphthalene acetic acid; *NM* not mentioned; *PP* photoperiod; *rpm* rotation per minute; *r*.*t*. room temperature; *SAM* shoot apical meristem; *TDZ* thidiazuron; *w* weeks

## Main text

### In vitro autopolyploidization

**Fig. 1:**
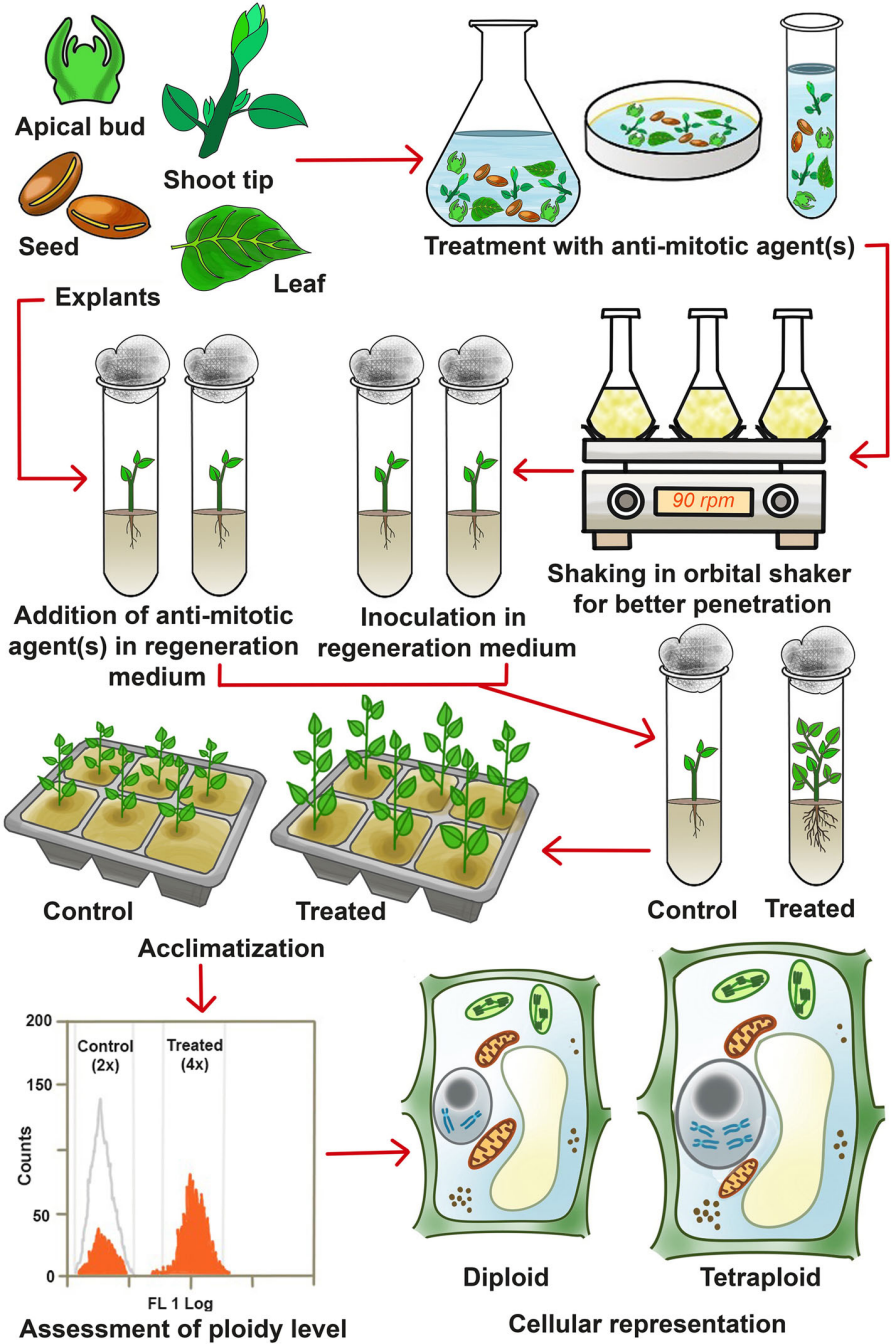
Diagrammatic representation of in vitro induction of autopolyploidization (diagram is not in scale) (Source: unpublished diagram of Saikat Gantait)

The general procedure of in vitro autopolyploidisation involves initial treatment of the explants with a suitable antimitotic agent followed by shaking the treated explants on an orbital shaker, before being inoculated into culture media. As an alternative approach, antimitotic agents could directly be added to the basal media containing the inoculated explants for possible polyploidization, although the earlier process is more effective and less time taking. Subsequent culture, growth, acclimatization and morphological assessment, along with confirmation of chromosome doubling through flow cytometry and chromosome counting, are performed accordingly (Fig. 1). The various factors affecting successful in vitro polyploidization are discussed in the following sections.

### Influence of treatment condition

Treatment conditions determine the final outcome of the experiment as they affect the endogenous metabolic processes leading to the upregulation or downregulation of the biochemical production. In most of the experiments, the explants were dipped, soaked or immersed into the polyploidy inducing solution resulting in imbibition of the chemicals. Colchicine, a polyploidy inducer, was applied after dissolving the same in dimethyl sulfoxide (DMSO) solution. Xing et al. [9] documented the enhanced effectiveness of colchicine dissolved in ethanol. The purpose of this was to increase the permeability of antimitotic agent(s) facilitating their entry into the regenerative tissues of the plant parts. As low as 2% (v/v) DMSO was most commonly used (Table 1). Similarly, oryzalin was reported to be dissolved in ethanol [55] or DMSO (1%) [62]. Exposure condition of the explants to antimitotic chemicals was recorded to differ across various publications. ‘Cotton ball or plug method’ for colchicine treatment was followed in several of the experiments (Table 1). Other experimental investigations state the addition of colchicine into the Murashige and Skoog (MS) basal medium [72] alone or in supplementation with plant growth regulators (PGRs), like α-naphthalene acetic acid (NAA), N^6^-benzyladenine (BA) or thidiazuron (TDZ). Inoculation in liquid MS basal media (MSL) supplemented with colchicine were also reported (Table 1). In addition, it was found in some reports that inoculation was carried out by means of constant shaking, using an orbital shaker. In most of the cases, shaking was done at 100 rpm. Presumably, use of liquid basal media supplemented with orbital shaking increased the absorption of antimitotic agents by further increasing the per unit surface area exposure over time. However, such prolonged durations may harm young explants for instance delicate meristematic meristems, which can be avoided with the aid of the most effective chemical-soaked cotton plugging method. Dark conditions during the colchicine inoculation period were adopted in several investigations (Table 1). Such shock treatment imposed on explants undergoing high cell division (callus, seed) promotes redifferentiative meristematic activity and germination and in process exposure to polyploidy inducers. Outstandingly, high temperature treatments resulting in the development of 2n female gametes for production of triploids was carried out in *Eucommia* sp. [53]. Increased temperature interferes with spindle formation and affects chromosome multiplication during the process.

### Influence of plant part(s)/explant

Appropriate selections of explants are imperative for effective polyploidization. The basic idea is that the explants must provide maximum conversion (polypoid cells) upon treatment that in turn would lead to generation of stable, high phytochemical yielding autopolyploid lines. Hence, young meristematic portions are more flexible to polyploidy induction, since they provide better permeability to the antimitotic chemicals; furthermore, as they contain fresh DNA as a result, duplication of genetic content becomes easier and more effective. Across the recently published research reports, it has been found that shoot tips, root tip, shoot apical meristem, petiole, nodal segments, callus, stem disc and leaf were successfully utilized as explants (Table 1). Seeds have also been used for the initiation of polyploidization experiments (Table 1). Other meristematic regions that were investigated into are terminal buds [60], adventitious buds [51], nodal buds [19], cluster buds [31] and axillary buds [55]. As a novel approach, diploid shooty teratomas obtained from *Agrobacterium tumefasciens-*mediated transformation were utilized for production of stable high vincristine, yielding tetraploid clones in *Catharanthus roseus* [11]. Clove explants in garlic were employed by Yousef and Elsadek [56]. Another unconventional explant that was utilized are protocorm-like bodies by [41] for tetraploid induction in *Dendrobium* hybrids. Tsuro et al. [32] have used callus for in vitro autoployploidy induction in *Chamaemelum nobile* L. Explant tissues should always receive uniform treatment otherwise it may lead to production of mixoploids having variable cytology [79]. Such results were found in the findings of Mansouri and Bagheri [36], although an increase in cannabinoid content was found in mixoploids over tetraploids. From the different results obtained, shoot tip explants were the most effective during enhancement of phyto-biochemicals in induced autoploids (2–4-fold on average), whereas seeds and apical buds yielded similar improvements (1.5–2.5 times). Such effectivity could be attributed to their higher meristematic activity. Other explant sources (callus, leaf, somatic embryos, etc.) showing redifferentiative regeneration presented 2–4-fold increment in phytochemicals but there remains a chance of occurrence of somaclonal variants and hence reproducibility is compromised. It must be borne in mind that ameliorated secondary metabolite extraction through induced polyploidy asks for uniform and homogenized phytochemical production and end-product realization. Divergent explants being in their divergent stages of physiological development ramify divergent responses. Differential responses of the explants engaged could be attributed to different factors such as physiological, morphological, biochemical or genetic factors [6]. Meristematic status as well as size of explants favours effective manipulation into desired experimental outcome [80]. Furthermore, different explants have different regenerative capabilities. Hence, apical meristems (shoot or root) with active cell divisional status are more amenable to ploidy alterations and in regeneration of autopolyploid shootlets. On the other hand, other explant sources (such as calli and somatic embryos) with varied tissue organizations might show experimental discrepancies into the realization of expected result.

### Influence of type, dosage and exposure duration of antimitotic agent(s)

Antimitotic agents are those group of chemicals that impede spindle formation during mitotic cell division, thereby resulting in the restitution of nucleus to contain all the chromosomes. As a result, the chromosome number of the cell doubles. Conversion to higher ploidy levels comprised of diploids is altered to triploids, tetraploids and octaploids. In the majority of the studies, the usage of colchicine as an antimitotic chemical was reported. However, other mitotic-toxic substances such as oryzalin, trifluralin were also put into use. Colchicine is a slow penetrating chemical impacting epidermal layer, subepidermal layer or entire tissue depending upon its duration of exposure. It mostly affects the dividing cells through interception of spindle fibre formations. Frequent treatments are applied at brief intermissions to juvenile tissues since only a limited number of cells remain at their dividing stage at a particular point of time. ‘Cotton plug’ method [81] involving intermittent colchicine application presents an instance to the above situation. This method is advantageous in the sense that the chemical saturated cotton preserves moisture and ensures better contact with the chemical thereby effecting better cellular interaction and prospective ploidy level changes. However, it must be ensured that the polyploidy inducing chemical is supplied regularly otherwise the explant might dry. Varied doses of colchicine were applied (0.01%, 0.05%, 0.1%, 0.2%, 0.25%, 0.3%, 0.5%, 0.6%, 1%, 1.5%, and 2.5%; w/v) for successful polyploidization (Table 1). Very lower (< 1%) concentrations of colchicine were less productive resulting in less frequent polyploidization, whereas higher (> 2%) concentrations exhibited detrimental effects on the exposed plant tissues. The most efficient dose of colchicine was recorded to be 0.1% in terms of higher frequency polyploid development and higher production of secondary metabolites from those polyploids as well. In comparison to colchicine, there are fewer reports utilizations of trifluralin, oryzalin or amiprophos-methyl as antimitotic agents for autopolyploid induction (Table 1). Even though these reports testified significant improvement in secondary metabolite productions in (trifluralin-, oryzalin- or amiprophos-methyl-induced) tetraploids (1.5–2-fold), but the enhancement were comparatively lower to that of the colchiploids (colchicine-induced polyploids) (1.5–4-fold on an average). Preferences for the colchicine as an antimitotic agent account for its better action than other polyploidy inducing chemicals (such as trifluralin, oryzalin and amiprophos-methyl) due its better penetrance and quicker mobilization through the tissues. A careful study of the published reports established the fact that in case of successful colchicine treatment, usually lower concentrations were accompanied with longer exposure duration and vice versa. The treatment durations ranged from min to days. Shortest treatment being 20–40 min in *Andrographis paniculata* [47]. However, such a short exposure intensity was supported via ‘pre-treatment soaking’ in sterile water and subsequent ‘low temperature shock’ (Table 1). Such predisposition presumably improved seed germination and in process exposed the protruded shoot apical meristems to colchicine. In terms of exposure time, the longest duration was recorded as 37 days in *Echinacea purpurea* L. [28], wherein colchicine was added to the basal media resulting in high frequency tetraploids and octaploids along with augmented cichoric acid production. Addition of antimitotic agents to the basal media increased the exposure time which resulted in higher ploidy level in individual plants. However, low doses of colchicine were added to the media so as to prevent long time exposure-related injury to the tissues. Le et al. [66] have reported initial inoculation in colchicine supplemented basal media for the respective treatment durations and subsequent culturing in fresh colchicine free basal media. The most promising treatment duration was 24 h (Table 1), wherein maximum yield of secondary metabolites was recovered. For the other antimitotic agents (such as trifluralin, oryzalin and amiprophos-methyl), treatment duration could not be determined due to lack of comprehensive investigation. Rahman [35] reported 120 h of trifluralin exposure in *Aquilaria malaccensis* Lamarck. resulting in production of tetraploids, revealing the presence of sesquiterpenes. Similarly, oryzalin treatment was done mostly for 24 h except for Pliankong et al. [34] wherein exposure for only 6 h in *Capsicum frutescens* L. resulted in tetraploids with elevated capsaicin levels. Different durations of treatments have shown to give different responses [80]. As discussed earlier, exposures of longer duration with lower doses of antimitotic agent have been more effective than exposures of shorter duration coupled with higher concentrations. Both the extremities of doses stand unprofitable either due to non-effectivity or lethality [61, 79]. Different experimental results testify to the corollary that varied autopolyploids like mixoploids and tetraploids have been obtained due differential durations of treatment [64]. Changes in secondary cellular metabolism inflicted due to varied colchicine durations were also reported in recent past [57, 63]. Such subtlety in results across the different published research investigations could be due to differences in chemical absorption in the different tissues of the explants over time and consequent chromosome multiplicative interactions. Differences in durations could also be accounted to explant variedness (size, meristematic tissue status, presence of external cell surface substances like cuticle, waxes, seed coat) and species dependent amenability to artificially induced ploidy changes [80].

### Influence on secondary metabolite production

**Fig. 2:**
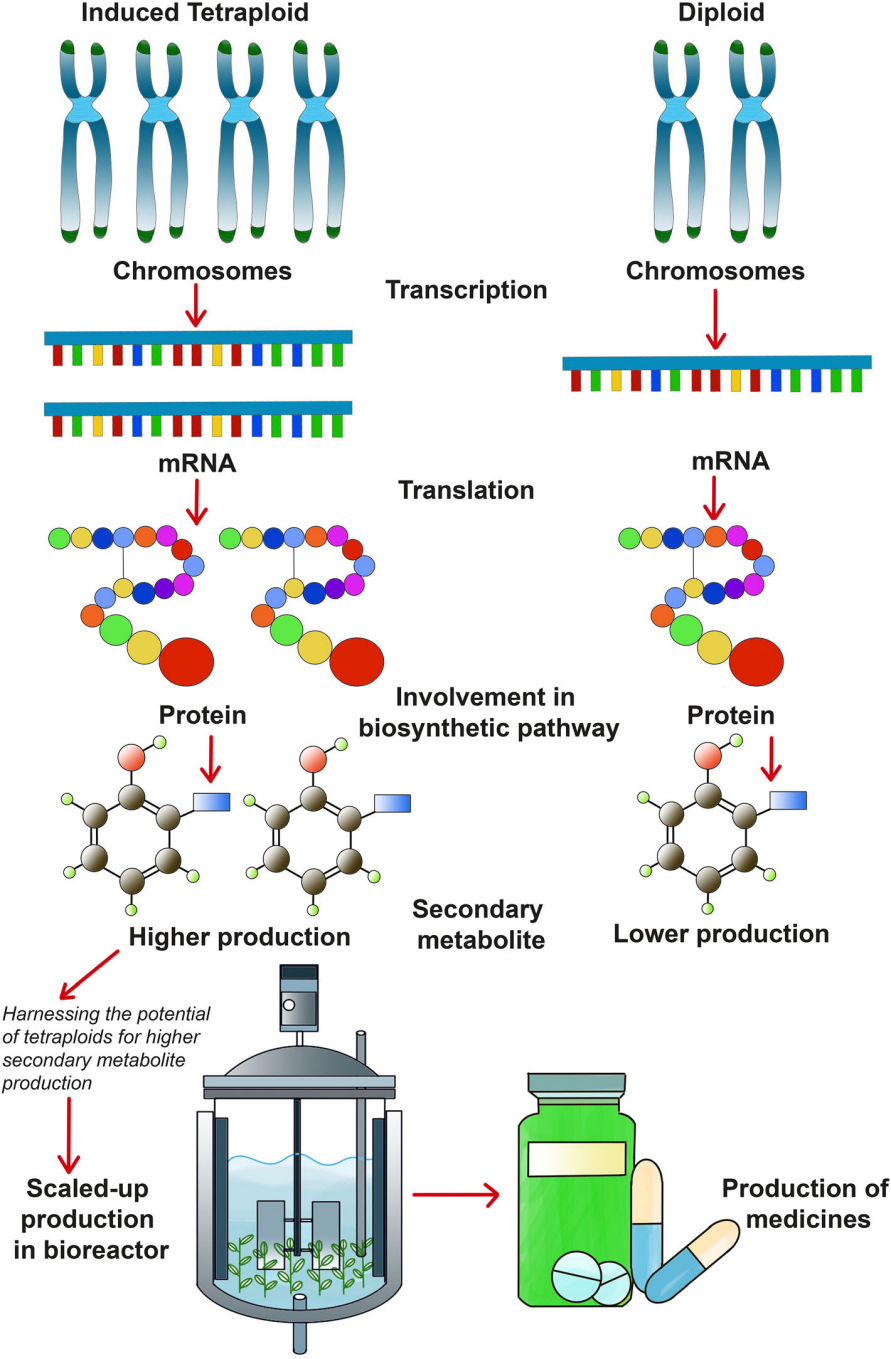
Diagrammatic representation of difference in gene expression in autopolyploids and diploids leading to differential secondary metabolite production (diagram is not in scale) (Source: unpublished diagram of Saikat Gantait)

In medicinal plants, secondary metabolites produced per unit biomass are of immense economic importance. Necessarily, in this scenario, doubling of the genomic content of cells propounds functionalized genome multiplications, enhanced protein synthesis and thereby amplified secondary metabolite biosynthesis among the induced autotetraploids (Fig. 2). Alterations in cellular dynamics is positively influenced owing to pronounced increase in cell size, organelle size and numbers, transcriptome products, net photosynthetic rate and upraised metabolistic pathways. Accumulation of favourable ‘like’ alleles in one organism coupled with induced doubling of chromosome number further aids in the pharma-chemical productivity and promotes partitioning of cell energy resources for secondary metabolism and trimming down of lengthy pathways via improvised enzyme kinetics [4]. This holds an evolutionary significance as energy saving plants are more favoured. Positive mutagenic changes like deletions, additions translocations or insertions restructure genomic architecture and invoke epigenetic permutations (DNA methylations, gene-silencing). Induced genome multiplications could also impinge cellular homeostatics due to imposed shock leading to inconsistent biochemical production [5, 82]. Table 1 represents a survey of available literature that reveal that there is an accrual in secondary metabolites production among the induced autopolyploids. Such amelioration brought about was recorded to be 2–4 times. Secondary metabolites targeted were alkaloids [52], phenolic acids like, cichoric acid [15], caffeic acid [22], terpenoids [10], glycosides [51], essential oils [27] and flavonoids [37]. However, findings of Caruso et al. [13] revealed that similar or lower quantities of phenylpropanoids and metabolites were produced in induced tetraploids of *Solanum bulbocastanum* in comparison to their diploid counterparts. These results differ from the same researcher’s previous findings in *Solanum commersonii* [12] wherein the increment in phenylpropanoid content (~ 2 times) was observed. Thus, such investigations establish the fact that species-related differences eventually affect polyploidy induction with respect to phytometabolite synthesis. Claims of lower production of phenolic compounds were found in the experimental reports of Ghimire et al. [29], wherein hexaploids of *Miscanthus* × *giganteus* yielded less than their corresponding triploids. However, it was observed that there was quantitative alteration in the component phenolic acids that were produced in the hexaploids as compared to the triploids. Supposedly, there was modification in the gene expression in hexaploids leading to repression and/or de-repression of genes for phenolic acid biosynthesis. Such quantitative and non-quantitative changes leading to biosynthesis of novel phytochemicals originally not detected in their diploid parents were also reported most recently [64, 65, 70]. Cara et al. [70] in their experimental findings documented comparative variations in the secondary biometabolite productions among induced autopolyploids and allopolyploids of *Solanum tuberosum* and *Solanum kurtzianum* (Table 1). Obtained altered secondary metabolism as exhibited in their results find relevance in stress resistance breeding due to identification and development of lines producing more amount of stress tolerance conferring osmolytes. Similar opinions of modulations in metabolism favouring plant species adaptability were opined by Kumar [82]. Li et al. [28] in their findings in *Echinacea purpurea* reported cichoric acid enhancement through tetraploid and subsequent octaploid induction. From the results, it can be deduced that the rate of increment in metabolite production increases in tetraploids and is further improved in octaploids. Hence, manifold multiplication of allelic constituents further improvises metabolism pathways through complementation in enzyme productions and action. Such polyploidy induction could be yet another remunerative approach for in vitro autopolyploidization-mediated crop improvement. However, the above-mentioned fact contradicts with opinions of Lavania et al. [4], wherein it was hypothesized that cytosine methylation in genomic regions in tandem with ploidy upliftment diminishes biometabolites production. Occasionally appearing mixoploids (organisms with differential chromosome numbers across cells; chimera) express enrichment of pharmaceutical metabolites as seen in *Stevia rebaudiana* (Bertoni) (13.5% increase in steviosides content) [24]. Similarly, Mansouri and Bagheri [36] in their study reported an increase in cannabinoids in induced mixoploids over tetraploids. Such results were due to decreased trichomes in tetraploids than mixoploids, hinting at probable negatively correlated gene interactions. In this context, Herawati et al. [69] reported increased numbers of glandular trichomes correlating positively with artemisinin production. Throughout the different studies, it can also be concluded that enhancement in bio-metabolites productions upon induced chromosome doubling is not exactly twice but the ameliorations are more than their diploid counterparts (Table 1). Improvements in secondary biometabolites productions could be due to morphological, physiological as well as genetic manifestations engendered upon by induced autoploids. Some of such illustrative causes of autoploidy-mediated secondary metabolite augmentation are increase in stomatal sizes and densities leading to increased photosynthates output [67], heterosis and gene redundancy imparting transgressive meliorism [56, 66, 68]. Shmeit et al. [62] reckoned that improvised primary metabolism could be the presumptive cause of ameliorated secondary metabolism. Modified enzyme profiles and gene expressions also play role in this refinement.

## Conclusions

Autopolyploidy is supposed to have an effect on the plant vigour in terms of biomass production and phytochemical biosynthesis. Proliferation in morphological traits and change in cytological structures coupled with novel proteomic features and accumulated secondary phyto-pharmaceuticals are major advantages established in induced autopolyploid cultures. Choice of suitable metaphase inhibitors or the polyploidy inducers is important as these are major players in the process. Of the different antimitotic agents, colchicine has proved to be better in producing stable and functional autoploids in higher frequencies. Potency of colchicine is due to its better cell to cell mobilization and tissue permeation, thereby resulting in more microtubule blockage during divisional stages and producing autopolyploids. Of the different explants, meristematic shoot tips exhibited higher ploidy conversions. Optimized results were obtained at 0.1% for 24 h of colchicine resulting in desired ploidy level manipulations and improved secondary metabolism. Improvements in biometabolite synthesis stems from improvised enzymatic functioning. Formulation of optimized protocol in different medicinal crops of value enables coordinated in vitro omics studies, contributes to crop evolution studies and unravels unsolved taxonomic queries. This tool in conjunction with next-generation sequencing techniques, hairy root culture technologies and other biotechnological interventions will certainly help researchers in future plant breeding strategies. The demand for medicinally valued plants for treatment and cure of different diseases has increased worldwide. Hence, keeping in mind about the commercial and scientific realizations, ameliorated secondary bio-metabolite production via in vitro induced autopolyploidy emerges as a constructive strategy for morpho-physio-genetic crop improvement.

## Data Availability

The datasets used and/or analyzed during the current study are available from the corresponding author on reasonable request.

## Abbreviations

BA N^6^: Benzyladenine
DMSO: Dimethyl sulfoxide
MS: Murashige and Skoog
MSL: MS liquid media
NAA: α-Naphthalene acetic acid
TDZ: Thidiazuron
